# Supplementary material for: Fluoroquinolone resistance determinants in carbapenem-resistant Escherichia coli isolated from urine clinical samples in Thailand
Source: PeerJ. 2023 Nov 8;11:e16401. doi: 10.7717/peerj.16401 (PMC10638923; doi:10.7717/peerj.16401)
Supplement: Supplemental Information 1 [file peerj-11-16401-s001.docx]

**Supplement data**


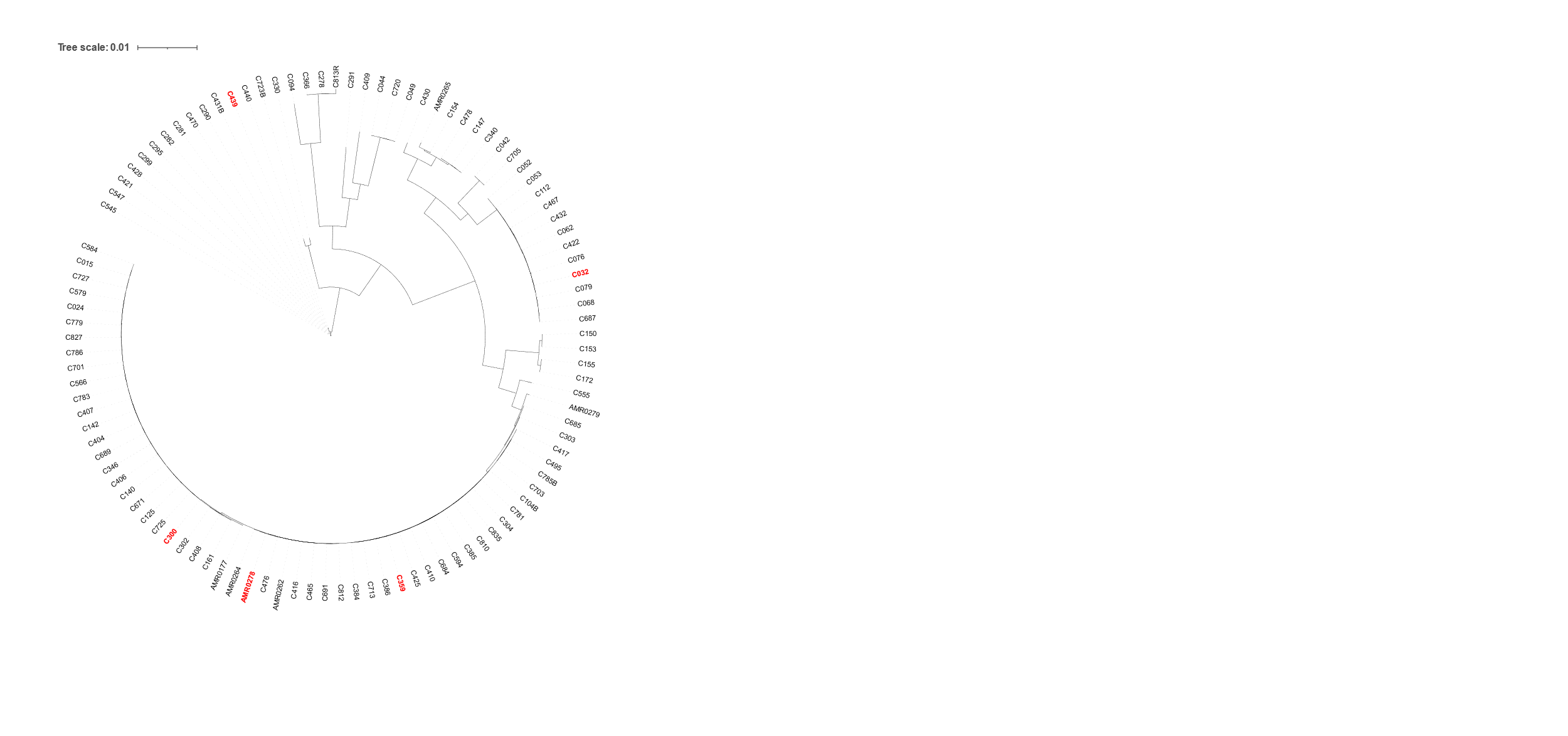


**Figure S1.** Core-genome maximum-likelihood phylogeny based on 103 CREc isolates genomes reconstructed using Panaroo and IQ-TREE. The tree was visualized using iToL.

**Table S1.** Genotypic profiles, fluoroquinolone susceptibility and ESBL production in 103 CREc from urine clinical samples.

| **Strain ID** | **Genome accession No.** | **MLST** | ***FimH*** | **Serotypes** | **Clermont phylogroup** | **Date collected** | **Reference** |
| --- | --- | --- | --- | --- | --- | --- | --- |
| C015 | DRX115310 | 410 | *fimH*24 | O8:H9 | C | 19/10/2015 | Takeuchi, et al., 2022 |
| C024 | DRX115317 | 410 | *fimH*24 | O8:H9 | C | 22/1/2016 | Takeuchi, et al., 2022 |
| C032 | JAPFQZ000000000 | 361 | *fimH*54 | O9:H30 | A | 29/1/2016 | Takeuchi, et al., 2022 |
| C042 | DRX115332 | 46 | Unknown type | O9:H10 | A | 6/3/2015 | Takeuchi, et al., 2022 |
| C044 | DRX115334 | 354 | *fimH*58 | O45:H6 | F | 21/3/2015 | Takeuchi, et al., 2022 |
| C049 | DRX115339 | 354 | *fimH*58 | O45:H6 | F | 29/4/2015 | Takeuchi, et al., 2022 |
| C052 | DRX115340 | 361 | *fimH*54 | O9:H30 | A | 25/8/2015 | Takeuchi, et al., 2022 |
| C053 | DRX187461 | 361 | *fimH*54 | O9:H30 | A | 23/8/2015 | Takeuchi, et al., 2022 |
| C062 | DRX115349 | 361 | *fimH*54 | O9:H30 | A | 5/1/2016 | Takeuchi, et al., 2022 |
| C068 | DRX115354 | 361 | *fimH*54 | O9:H30 | A | 16/3/2016 | Takeuchi, et al., 2022 |
| C079 | DRX115365 | 361 | *fimH*54 | O9:H30 | A | 20/2/2016 | Takeuchi, et al., 2022 |
| C094 | DRX115374 | 1193 | *fimH*64 | O75:H5 | B2 | 19/4/2016 | Takeuchi, et al., 2022 |
| C112 | DRX187454 | 361 | *fimH*54 | O9:H30 | A | 3/5/2016 | Takeuchi, et al., 2022 |
| C125 | DRX115403 | 410 | *fimH*24 | O8:H9 | C | 25/4/2016 | Takeuchi, et al., 2022 |
| C140 | DRX115416 | 410 | *fimH*24 | O8:H9 | C | 7/7/2016 | Takeuchi, et al., 2022 |
| C142 | DRX115418 | 410 | *fimH*24 | O8:H9 | C | 4/8/2016 | Takeuchi, et al., 2022 |
| C147 | DRX115422 | 167 | Unknown type | O89:H5 | A | 6/8/2016 | Takeuchi, et al., 2022 |
| C153 | DRX141043 | 448 | *fimH*35 | H8 | B1 | 12/8/2016 | Takeuchi, et al., 2022 |
| C154 | DRX141044 | 167 | Unknown type | O89:H5 | A | 16/8/2016 | Takeuchi, et al., 2022 |
| C161 | DRX141051 | 410 | *fimH*24 | O8:H9 | C | 27/5/2015 | Takeuchi, et al., 2022 |
| C278 | DRX187483 | 131 | *fimH*30 | O25:H4 | B2 | 23/3/2016 | Takeuchi, et al., 2022 |
| C281 | DRX187484 | 405 | *fimH*29 | O102:H6 | D | 7/4/2016 | Takeuchi, et al., 2022 |
| C282 | DRX141151 | 405 | *fimH*29 | O102:H6 | D | 6/4/2016 | Takeuchi, et al., 2022 |
| C290 | DRX187485 | 405 | *fimH*29 | O102:H6 | D | 26/6/2016 | Takeuchi, et al., 2022 |
| C291 | DRX141161 | 2011 | *fimH*27 | O1:H6 | F | 30/6/2016 | Takeuchi, et al., 2022 |
| C295 | DRX141165 | 405 | *fimH*29 | O102:H6 | D | 29/7/2016 | Takeuchi, et al., 2022 |
| C300 | JAPFQY000000000 | 410 | *fimH*24 | O8:H9 | C | 18/9/2016 | Takeuchi, et al., 2022 |
| C302 | DRX141171 | 410 | *fimH*24 | O8:H9 | C | 8/10/2016 | Takeuchi, et al., 2022 |
| C303 | DRX141172 | 410 | *fimH*24 | H9 | C | 3/10/2016 | Takeuchi, et al., 2022 |
| C304 | DRX141173 | 10210 | *fimH*24 | H9 | C | 13/10/2016 | Takeuchi, et al., 2022 |
| C340 | DRX141206 | 167 | Unknown type | O89:H5 | A | 5/11/2016 | Takeuchi, et al., 2022 |
| C346 | DRX141211 | 410 | *fimH*24 | O8:H9 | C | 10/11/2016 | Takeuchi, et al., 2022 |
| C359 | JAPFQX000000000 | 410 | *fimH*24 | H9 | C | 2/10/2016 | Takeuchi, et al., 2022 |
| C384 | DRX141247 | 410 | *fimH*24 | H9 | C | 4/10/2016 | Takeuchi, et al., 2022 |
| C385 | DRX141248 | 410 | *fimH*24 | H9 | C | 11/10/2016 | Takeuchi, et al., 2022 |
| C386 | DRX141249 | 410 | *fimH*24 | H9 | C | 11/10/2016 | Takeuchi, et al., 2022 |
| C404 | DRX141269 | 410 | *fimH*24 | O8:H9 | C | 17/11/2016 | Takeuchi, et al., 2022 |
| C406 | DRX141271 | 410 | *fimH*24 | O8:H9 | C | 20/11/2016 | Takeuchi, et al., 2022 |
| C407 | DRX141272 | 410 | *fimH*24 | O8:H9 | C | 20/11/2016 | Takeuchi, et al., 2022 |
| C408 | DRX141273 | 410 | *fimH*24 | O8:H9 | C | 23/11/2016 | Takeuchi, et al., 2022 |
| C409 | DRX141274 | 457 | *fimH*145 | O11:H25 | F | 23/11/2016 | Takeuchi, et al., 2022 |
| C410 | DRX141275 | 410 | *fimH*24 | H9 | C | 24/11/2016 | Takeuchi, et al., 2022 |
| C417 | DRX141282 | 410 | *fimH*24 | H9 | C | 26/11/2016 | Takeuchi, et al., 2022 |
| C421 | DRX141285 | 405 | *fimH*29 | O102:H6 | D | 6/9/2016 | Takeuchi, et al., 2022 |
| C422 | DRX141286 | 361 | *fimH*54 | O9:H30 | A | 7/9/2016 | Takeuchi, et al., 2022 |
| C430 | DRX141293 | 34 | *fimH*54 | O101:H33 | A | 14/10/2016 | Takeuchi, et al., 2022 |
| C432 | DRX141296 | 361 | *fimH*54 | O9:H30 | A | 19/10/2016 | Takeuchi, et al., 2022 |
| C439 | JAPJYK000000000 | 405 | *fimH*29 | O102:H6 | D | 11/11/2016 | Takeuchi, et al., 2022 |
| C465 | DRX141307 | 410 | *fimH*24 | H9 | C | 24/12/2016 | Takeuchi, et al., 2022 |
| C470 | DRX187488 | 405 | *fimH*29 | O102:H6 | D | 10/1/2017 | Takeuchi, et al., 2022 |
| C476 | DRX141318 | 410 | *fimH*24 | H9 | C | 12/1/2017 | Takeuchi, et al., 2022 |
| C478 | DRX141320 | 167 | Unknown type | O89:H5 | A | 10/1/2017 | Takeuchi, et al., 2022 |
| C495 | DRX141333 | 410 | *fimH*24 | H9 | C | 24/1/2017 | Takeuchi, et al., 2022 |
| C545 | DRX141349 | 405 | *fimH*29 | O102:H6 | D | 12/1/2017 | Takeuchi, et al., 2022 |
| C547 | DRX141351 | 405 | *fimH*29 | O102:H6 | D | 27/1/2017 | Takeuchi, et al., 2022 |
| C555 | DRX141359 | 88 | *fimH*43 | H12 | C | 17/3/2017 | Takeuchi, et al., 2022 |
| C566 | DRX141365 | 410 | *fimH*24 | O8:H9 | C | 5/4/2017 | Takeuchi, et al., 2022 |
| C579 | DRX141376 | 410 | *fimH*24 | O8:H9 | C | 4/4/2017 | Takeuchi, et al., 2022 |
| C584 | DRX141381 | 410 | *fimH*24 | O8:H9 | C | 16/3/2017 | Takeuchi, et al., 2022 |
| C594 | DRX141391 | 410 | *fimH*24 | H9 | C | 17/1/2017 | Takeuchi, et al., 2022 |
| C671 | DRX141416 | 410 | *fimH*24 | O8:H9 | C | 12/4/2017 | Takeuchi, et al., 2022 |
| C685 | DRX141427 | 410 | *fimH*24 | H9 | C | 14/5/2017 | Takeuchi, et al., 2022 |
| C687 | DRX141429 | 361 | *fimH*54 | O9:H30 | A | 16/5/2017 | Takeuchi, et al., 2022 |
| C689 | DRX141431 | 410 | *fimH*24 | O8:H9 | C | 3/5/2017 | Takeuchi, et al., 2022 |
| C701 | DRX141443 | 410 | *fimH*24 | O8:H9 | C | 15/5/2017 | Takeuchi, et al., 2022 |
| C703 | DRX141445 | 410 | *fimH*24 | H9 | C | 12/5/2017 | Takeuchi, et al., 2022 |
| C705 | DRX141447 | 46 | Unknown type | O9:H10 | A | 28/3/2017 | Takeuchi, et al., 2022 |
| C713 | DRX141455 | 410 | *fimH*24 | H9 | C | 20/4/2017 | Takeuchi, et al., 2022 |
| C720 | DRX141462 | 354 | *fimH*58 | O45:H6 | F | 3/5/2017 | Takeuchi, et al., 2022 |
| C725 | DRX141468 | 410 | *fimH*24 | O8:H9 | C | 18/5/2017 | Takeuchi, et al., 2022 |
| C727 | DRX141470 | 410 | *fimH*24 | O8:H9 | C | 22/5/2017 | Takeuchi, et al., 2022 |
| C781 | DRX141473 | 10210 | *fimH*24 | H9 | C | 30/5/2017 | Takeuchi, et al., 2022 |
| C783 | DRX141475 | 410 | *fimH*24 | O8:H9 | C | 20/5/2017 | Takeuchi, et al., 2022 |
| C786 | DRX141480 | 410 | *fimH*24 | O8:H9 | C | 6/6/2017 | Takeuchi, et al., 2022 |
| C810 | DRX141503 | 410 | *fimH*24 | H9 | C | 31/5/2017 | Takeuchi, et al., 2022 |
| C812 | DRX141505 | 410 | *fimH*24 | H9 | C | 12/6/2017 | Takeuchi, et al., 2022 |
| C827 | DRX141510 | 410 | *fimH*24 | O8:H9 | C | 18/7/2017 | Takeuchi, et al., 2022 |
| C835 | DRX141518 | 410 | *fimH*24 | H9 | C | 21/7/2017 | Takeuchi, et al., 2022 |
| C104B | DRX115384 | 410 | *fimH*24 | H9 | C | 4/6/2016 | Takeuchi, et al., 2022 |
| C431W | DRX141295 | 405 | *fimH*27 | O102:H6 | D | 17/10/2016 | Takeuchi, et al., 2022 |
| C723B | DRX141465 | 38 | Unknown type | O86:H18 | D | 15/5/2017 | Takeuchi, et al., 2022 |
| C785B | DRX141478 | 410 | *fimH*24 | H9 | C | 8/6/2017 | Takeuchi, et al., 2022 |
| C172 | DRX141063 | 448 | *fimH*35 | H8 | B1 | 4/8/2015 | Takeuchi, et al., 2022 |
| C330 | DRX141198 | 38 | *fimH*65 | O1:H15 | D | 5/9/2016 | Takeuchi, et al., 2022 |
| C416 | DRX141281 | 10210 | *fimH*24 | H9 | C | 22/11/2016 | Takeuchi, et al., 2022 |
| C467 | DRX141309 | 361 | *fimH*54 | O9:H30 | A | 9/11/2016 | Takeuchi, et al., 2022 |
| C684 | DRX141426 | 410 | *fimH*24 | H9 | C | 8/5/2017 | Takeuchi, et al., 2022 |
| C779 | DRX141471 | 410 | *fimH*24 | O8:H9 | C | 2/6/2017 | Takeuchi, et al., 2022 |
| AMR0177 | JAPIWS000000000 | 410 | *fimH*24 | O51:H9 | C | 11/6/2020 | This study |
| AMR0262 | JAPIWR000000000 | 410 | *fimH*24 | H9 | C | 14/5/2020 | This study |
| C155 | JAPDNK000000000 | 448 | *fimH*35 | H8 | B1 | 10/2/2015 | This study |
| C440 | JAPIWN000000000 | 405 | *fimH*29 | O102:H6 | D | 13/11/2016 | This study |
| AMR0264 | JAPIWQ000000000 | 410 | *fimH*24 | H9 | C | 16/5/2020 | This study |
| C076 | JAPDNJ000000000 | 361 | *fimH*54 | O9:H30 | A | 3/2/2016 | This study |
| AMR0265 | JAPIWP000000000 | 1702 | Unknown type | O89:H9 | A | 17/5/2020 | This study |
| AMR0279 | JAPIWO000000000 | 410 | *fimH*24 | O8:H17 | C | 28/5/2020 | This study |
| C366 | JAPDNI000000000 | 131 | *fimH*30 | O25:H4 | B2 | 3/11/2016 | This study |
| C425 | JAPDNH000000000 | 410 | *fimH*24 | H9 | C | 17/9/2016 | This study |
| C691 | JAPDND000000000 | 410 | *fimH*24 | H9 | C | 3/5/2017 | This study |
| C428 | JAPDNE000000000 | 405 | *fimH*29 | O102:H6 | D | 26/9/2016 | This study |
| C150 | JAPDNG000000000 | 448 | *fimH*35 | H8 | B1 | 26/8/2016 | This study |
| C299 | JAPDNF000000000 | 405 | *fimH*29 | O102:H6 | D | 2/8/2016 | This study |
| AMR0278 | JAPDNL000000000 | 410 | *fimH*24 | H9 | C | 27/5/2020 | This study |

Abbreviation. MLST, multilocus sequence typing

**Table S2.** The quality assemblies of 5 FQ-CREc urine isolates in Thailand.

| **Strain ID** | **Total Sequence Length (bp)** | **Mean length** | **N50 (bp)** | **GCcontent (%)** |
| --- | --- | --- | --- | --- |
| C032 | 5070655 | 309 | 4812743 | 50.7 |
| C300 | 4877879 | 313.1 | 4767522 | 50.6 |
| C359 | 4924245 | 312 | 4832244 | 50.6 |
| C439 | 5284775 | 315 | 5026347 | 50.6 |
| AMR0278 | 5360354 | 305.1 | 4888115 | 50.6 |
| Mean | 5103581.6 | 310.84 | 4865394.2 | 50.62 |

| **Virulence factor classes** | **Virulence factors** | **Prevalence (%)** | **No. of positive/No. of total** |
| --- | --- | --- | --- |
| Adhesion | *fim* | 100 | 103/103 |
|  | *ecp* | 98.06 | 101/103 |
|  | *csg* | 100 | 103/103 |
|  | *eae* | 78.64 | 81/103 |
|  | *pap* | 21.36 | 22/103 |
|  | *sfa* | 0 | 0/103 |
|  | *afa,draP* | 3.88 | 4/103 |
|  | *aap* | 0.97 | 1/103 |
|  | *cfa* | 6.8 | 7/103 |
|  | *aaf* | 2.91 | 3/103 |
|  | *fae* | 1.94 | 2/103 |
| Invasion | *IbeABC* | 100 | 103/103 |
|  | *tia* | 49.51 | 51/103 |
| Toxin | *hlyE/clyA* | 97.09 | 100/103 |
|  | *senB* | 4.85 | 5/103 |
|  | *sat* | 3.88 | 4/103 |
|  | *astA* | 2.91 | 3/103 |
|  | *usp* | 2.91 | 3/103 |
|  | *HlyABD* | 0.97 | 1/103 |
|  | *CNF1* | 0.97 | 1/103 |
| Iron uptake | *fyuA* | 47.57 | 49/103 |
|  | *chuA* | 21.36 | 22/103 |
|  | *sitA* | 24.27 | 25/103 |
|  | *iucC* | 8.74 | 9/103 |
| Autotransporters | *upaG/ehaG* | 65.05 | 67/103 |
|  | *espl* | 97.09 | 100/103 |
|  | *ehaB* | 28.16 | 29/103 |
|  | *cah* | 67.96 | 70/103 |
|  | *agn43* | 15.53 | 16/103 |
|  | *cdi* | 10.68 | 11/103 |
|  | *pic* | 2.91 | 3/103 |
|  | *ehaA* | 3.88 | 4/103 |
|  | *vat* | 0.97 | 1/103 |
|  | *espC* | 0.97 | 1/103 |
|  | *aatA* | 0.97 | 1/103 |
|  | *upaH* | 0.97 | 1/103 |
| Protectin | *ompT* | 6.8 | 7/103 |

**Table S3.** Genotypic of 37 virulence factors of 103 urinary CRE

**Table S4.** Virulence factor classes in phylogenetic groups of 103 FQ-CREc urine isolates in Thailand.

| **Clermont phylogroup** | | **n** | | **Adhesion-associated virulence factors** | | | | | | | | | | | | | | | | | | | | | | | | | **Invasion** | | | | | **Toxin virulence factors** | | | | | | | | | | | | | | | **Iron uptake** | | | | | | | | | **Autotransporters** | | | | | | | | | | | | | | | | | | | | | | | | |  | |
| --- | --- | --- | --- | --- | --- | --- | --- | --- | --- | --- | --- | --- | --- | --- | --- | --- | --- | --- | --- | --- | --- | --- | --- | --- | --- | --- | --- | --- | --- | --- | --- | --- | --- | --- | --- | --- | --- | --- | --- | --- | --- | --- | --- | --- | --- | --- | --- | --- | --- | --- | --- | --- | --- | --- | --- | --- | --- | --- | --- | --- | --- | --- | --- | --- | --- | --- | --- | --- | --- | --- | --- | --- | --- | --- | --- | --- | --- | --- | --- | --- | --- | --- | --- | --- |
|  |  |  |  | ***fim*** | | ***ecp*** | | ***csg*** | | ***eae*** | | ***pap*** | | ***sfa*** | | ***afa, draP*** | | ***aap*** | | ***cfa*** | | ***aaf*** | | ***fae*** | | ***Ibe ABC*** | | ***tia*** | | | ***hlyE/***  ***clyA*** | | ***senB*** | | | ***sat*** | | ***astA*** | | ***usp*** | | ***HlyABD*** | | ***CNF1*** | | ***fyuA*** | | ***chuA*** | | | ***sitA*** | | ***iucC*** | | ***upaG/ehaG*** | ***espl*** | | | ***ehaB*** | | ***cah*** | | ***agn43*** | | ***cdi*** | | ***pic*** | | ***ehaA*** | | ***vat*** | | ***espC*** | | ***aatA*** | | ***upaH*** | | ***ompT*** | | **Mean** | | |  |
| C | 56 | | 100 | | 100 | | 100 | | 78.6 | | 3.57 | | 0 | | 0 | | 0 | | 7.14 | | 0 | | 1.8 | | 100 | | 32.1 | | | 100 | | 1.79 | | | 0 | | 0 | | 0 | | 0 | | 0 | | 41.1 | | 0 | | | 5.4 | | 5.4 | | 92.8 | | | 100 | | | 0 | | 94.6 | | 5.36 | | 0 | | 0 | | 0 | | 0 | | 0 | | 0 | | 0 | | 3.57 | | 26.30 | | |
| A | 20 | | 100 | | 100 | | 100 | | 75 | | 0 | | 0 | | 0 | | 0 | | 5 | | 0 | | 0 | | 100 | | 80 | | | 100 | | 5 | | | 0 | | 0 | | 0 | | 0 | | 0 | | 15 | | 0 | | | 45 | | 5 | | 60 | | | 100 | | | 75 | | 15 | | 5 | | 0 | | 0 | | 0 | | 0 | | 0 | | 0 | | 0 | | 0 | | 26.62 | | |
| D | 15 | | 100 | | 93.33 | | 100 | | 86.7 | | 93.3 | | 0 | | 13 | | 7 | | 0 | | 6.67 | | 6.7 | | 100 | | 93.3 | | | 100 | | 13.3 | | | 20 | | 13 | | 0 | | 0 | | 0 | | 100 | | 100 | | | 73 | | 20 | | 6.66 | | | 100 | | | 33.3 | | 46.7 | | 60 | | 67 | | 6.7 | | 0 | | 0 | | 6.7 | | 6.67 | | 0 | | 0 | | 39.82 | | |
| F | 5 | | 100 | | 100 | | 100 | | 100 | | 60 | | 0 | | 0 | | 0 | | 20 | | 0 | | 0 | | 100 | | 20 | | | 100 | | 0 | | | 0 | | 20 | | 0 | | 0 | | 0 | | 80 | | 100 | | | 0 | | 0 | | 0 | | | 100 | | | 80 | | 40 | | 0 | | 0 | | 0 | | 0 | | 0 | | 0 | | 0 | | 0 | | 40 | | 31.35 | | |
| B2 | 3 | | 100 | | 66.6 | | 100 | | 33.3 | | 100 | | 0 | | 0 | | 0 | | 0 | | 0 | | 0 | | 100 | | 33.3 | | | 0 | | 33.3 | | | 33 | | 0 | | 100 | | 33.3 | | 33.3 | | 66.7 | | 66.6 | | | 67 | | 67 | | 33.3 | | | 0 | | | 33.3 | | 33.3 | | 100 | | 33 | | 67 | | 0 | | 33 | | 0 | | 0 | | 33.3 | | 100 | | 40.54 | | |
| B1 | 4 | | 100 | | 100 | | 100 | | 75 | | 0 | | 0 | | 50 | | 0 | | 25 | | 50 | | 0 | | 100 | | 25 | | | 100 | | 0 | | | 0 | | 0 | | 0 | | 0 | | 0 | | 50 | | 0 | | | 0 | | 0 | | 25 | | | 100 | | | 100 | | 100 | | 0 | | 0 | | 0 | | 100 | | 0 | | 0 | | 0 | | 0 | | 0 | | 32.43 | | |

**Table S5** Antimicrobial resistance determinants, biofilm formation and plasmid replicon types of 103 FQ-CREc urine isolates in Thailand.

| **Codes** | **Antimicrobial resistance determinants** | | | | | | **Biofilm formations** | **Plasmid replicon types** |
| --- | --- | --- | --- | --- | --- | --- | --- | --- |
|  | **ESBL** | **CPFX** | **CPFX MIC** | **LVFX** | **LVFX**  **MIC** | **NFT** |  |  |
| C015 | ESBL | R | 32 | R | 16 | R | None | IncFIB(AP001918)_1,IncY_1,IncFIA_1,Col(BS512)_1,IncFII(pAMA1167-NDM-5)_1_pAMA1167-NDM-5 |
| C024 | ESBL | R | 32 | R | >32 | S | None | IncN2_1,IncFIB(AP001918)_1,IncFIA_1,IncFII(pAMA1167-NDM-5)_1_pAMA1167-NDM-5,Col(BS512)_1 |
| C032 | Non ESBL | R | 32 | R | >32 | S | None | IncFIB(AP001918),IncFII,IncFII(pRSB107),IncI(Gamma) |
| C042 | ESBL | R | 32 | R | >32 | S | None | ColRNAI_1,IncFII(pRSB107)_1_pRSB107,IncFIB(AP001918)_1,IncFIA_1 |
| C044 | ESBL | R | 32 | R | >32 | S | Strong | IncN2_1, IncFIA_1, IncFIB(pB171)_1_pB171, IncFII_1, Col8282_1 |
| C049 | ESBL | R | 32 | R | >32 | S | Moderate | IncFII_1, Col8282_1, IncFIB(pB171)_1_pB171, IncFIA_1, IncN2_1 |
| C052 | Non ESBL | R | 32 | R | >32 | S | None | Col(BS512)_1, Col440I_1,IncFII_1,IncFII(pRSB107)_1_pRSB107,IncI_Gamma_1 |
| C053 | Non ESBL | R | 32 | R | >32 | S | None | IncFII_1,IncFII(pRSB107)_1_pRSB107,IncI_Gamma_1,Col440I_1 |
| C062 | Non ESBL | R | 32 | R | >32 | S | None | IncI_Gamma_1,IncFIB(AP001918)_1,IncFII(pRSB107)_1_pRSB107,IncFII_1,Col440I_1 |
| C068 | Non ESBL | R | 32 | R | >32 | S | None | IncI_Gamma_1,IncFII(pRSB107)_1_pRSB107,IncFII_1,IncFIB(AP001918)_1,Col440I_1 |
| C079 | Non ESBL | R | 32 | R | >32 | S | None | IncFII_1,IncFII(pRSB107)_1_pRSB107,Col440I_1,IncI_Gamma_1,IncFIB(AP001918)_1 |
| C094 | Non ESBL | R | 32 | R | 12 | S | Moderate | IncX1_4,Col156_1,Col(VCM04)_1,IncA/C2_1,Col(BS512)_1,ColRNAI_1 |
| C112 | Non ESBL | R | 32 | R | >32 | S | None | IncFII(pRSB107)_1_pRSB107, pENTAS02_1, IncI_Gamma_1, Col440I_1, IncFIB(AP001918)_1, IncFII_1 |
| C125 | ESBL | R | 32 | R | >32 | S | None | IncFII(pAMA1167-NDM-5)_1_pAMA1167-NDM-5, Col(BS512)_1, IncX4_1, IncI1_1_Alpha, IncY_1, IncFIA_1, IncFIB(AP001918)_1 |
| C140 | ESBL | R | 32 | R | >32 | R | Strong | IncFIA_1,IncFIB(AP001918)_1,Col(BS512)_1,IncFII(pAMA1167-NDM-5)_1_pAMA1167-NDM-5 |
| C142 | ESBL | R | 32 | R | >32 | I | Strong | Col(BS512)_1,IncFIA_1,IncFIB(AP001918)_1,IncFII(pAMA1167-NDM-5)_1_pAMA1167-NDM-5 |
| C147 | ESBL | R | 32 | R | >32 | S | Weak | IncFIA_1,Col(BS512)_1,IncFIC(FII)_1,Col440I_1 |
| C153 | ESBL | R | 32 | R | 24 | S | Weak | IncFIA_1,IncFII(pAMA1167-NDM-5)_1_pAMA1167-NDM-5,IncFIB(AP001918)_1,IncI1_1_Alpha,IncX3_1,IncB/O/K/Z_2 |
| C154 | ESBL | R | 32 | R | >32 | S | Strong | IncI1_1_Alpha,Col(MG828)_1,IncFII_1 |
| C161 | ESBL | R | 32 | R | >32 | S | None | IncL/M(pMU407)_1_pMU407,IncX3_1,IncFIB(AP001918)_1,IncFIA_1,IncFII(pAMA1167-NDM-5)_1_pAMA1167-NDM-5,ColKP3_1 |
| C278 | ESBL | R | 32 | R | >32 | S | Moderate | IncFIB(AP001918)_1, IncFIC(FII)_1 |
| C281 | ESBL | R | 32 | R | >32 | S | Weak | IncFIB(AP001918)_1,IncFIA_1,IncFII(pRSB107)_1_pRSB107,Col(MG828)_1,Col(BS512)_1,Col440I_1 |
| C282 | ESBL | R | 32 | R | >32 | S | None | IncFII(pRSB107)_1_pRSB107,IncFIA_1,IncFIB(AP001918)_1,Col440I_1,Col(MG828)_1 |
| C290 | ESBL | R | 32 | R | >32 | S | None | Col(BS512)_1,IncFIB(AP001918)_1,IncFIA_1,IncFII(pRSB107)_1_pRSB107,Col440I_1,Col(MG828)_1 |
| C291 | Non ESBL | R | 32 | R | 16 | S | Moderate | ColRNAI_1,IncFIC(FII)_1,IncFIB(pB171)_1_pB171,Col8282_1,IncA/C2_1,Col(MG828)_1 |
| C295 | ESBL | R | 32 | R | >32 | S | Weak | Col(MG828)_1,IncFIB(AP001918)_1,IncFIA_1,IncFII(pRSB107)_1_pRSB107,Col(BS512)_1,ColRNAI_1,Col440I_1 |
| C300 | ESBL | R | 32 | R | 24 | R | None | Col(BS512),IncFIA,IncFIB(AP001918),IncFII(pAMA1167-NDM-5),IncQ1 |
| C302 | ESBL | R | 32 | R | >32 | I | Strong | IncA/C2_1,IncFII_1,IncFIB(AP001918)_1,IncFIA_1,IncFII(pAMA1167-NDM-5)_1_pAMA1167-NDM-5,IncN2_1,Col(BS512)_1,IncX1_4 |
| C303 | ESBL | R | 32 | R | >32 | R | None | IncFIB(AP001918)_1, IncFIA_1,IncA/C2_1, Col(BS512)_1, Col156_1 |
| C304 | ESBL | R | 32 | R | 16 | S | None | IncY_1, IncFIA_1, IncFII(pAMA1167-NDM-5)_1_pAMA1167-NDM-5, Col(BS512)_1, Col(IMGS31)_1,IncFIB(AP001918)_1 |
| C340 | ESBL | R | 32 | R | >32 | S | weak biofilm producer | Col(BS512)_1,IncFIC(FII)_1,Col440I_1,IncFIA_1 |
| C346 | ESBL | R | 32 | R | >32 | R | Strong | IncFIA_1,Col(BS512)_1,IncFIB(AP001918)_1,IncFII(pAMA1167-NDM-5)_1_pAMA1167-NDM-5 |
| C359 | ESBL | R | 32 | R | 24 | S | None | Col(BS512),IncFIA,IncFIB(AP001918),IncFII(pAMA1167-NDM-5) |
| C384 | ESBL | R | 32 | R | >32 | R | None | IncFIA_1,Col(BS512)_1,IncFIB(AP001918)_1,IncFII(pAMA1167-NDM-5)_1_pAMA1167-NDM-5 |
| C385 | ESBL | R | 32 | R | >32 | R | None | Col440I_1,Col440II_1,IncFIB(AP001918)_1,IncFIA_1,IncFII(pAMA1167-NDM-5)_1_pAMA1167-NDM-5,Col(BS512)_1 |
| C386 | ESBL | R | 32 | R | >32 | R | None | IncFII(pAMA1167-NDM-5)_1_pAMA1167-NDM-5,IncFIB(AP001918)_1,IncFIA_1,Col(BS512)_1 |
| C404 | ESBL | R | 32 | R | >32 | R | Strong | IncFIA_1, IncX1_4, IncFII_1, IncFIB(AP001918)_1, Col(BS512)_1, IncFII(pAMA1167-NDM-5)_1_pAMA1167-NDM-5 |
| C406 | ESBL | R | 32 | R | >32 | R | Strong | IncFIB(AP001918)_1, IncFII(pAMA1167-NDM-5)_1_pAMA1167-NDM-5, IncFIB(AP001918)_1, Col(BS512)_1 |
| C407 | ESBL | R | 32 | R | >32 | R | Strong | Col(BS512)_1,IncFIA_1,IncFII(pAMA1167-NDM-5)_1_pAMA1167-NDM-5,IncFIB(AP001918)_1 |
| C408 | ESBL | R | 32 | R | >32 | S | Strong | IncP1_3,IncN2_1,IncA/C2_1,IncFIB(AP001918)_1,IncFIA_1,Col(BS512)_1,IncX1_4,IncFII(pAMA1167-NDM-5)_1_pAMA1167-NDM-5,IncFII_1 |
| C409 | ESBL | R | 32 | R | 12 | S | Moderate | IncI1_1_Alpha,Col440II_1,ColRNAI_1,IncN2_1,IncFIB(AP001918)_1,ColRNAI_1,IncFIC(FII)_1 |
| C410 | ESBL | R | 32 | R | >32 | R | None | IncFIA_1,IncFIB(AP001918)_1,Col(BS512)_1,IncFII(pAMA1167-NDM-5)_1_pAMA1167-NDM-5 |
| C417 | ESBL | R | 32 | R | >32 | S | None | IncFIA_1,IncFIB(AP001918)_1,IncY_1,IncFII(pAMA1167-NDM-5)_1_pAMA1167-NDM-5,Col(BS512)_1,Col440I_1,Col440II_1 |
| C421 | ESBL | R | 32 | R | >32 | S | None | IncFII(pRSB107)_1_pRSB107,IncFIA_1,IncFIB(AP001918)_1,Col(MG828)_1,Col440I_1,Col(BS512)_1 |
| C422 | Non ESBL | R | 32 | R | >32 | S | None | IncFII(pRSB107)_1_pRSB107,IncI_Gamma_1,IncFIB(AP001918)_1,IncFII_1,Col440I_1 |
| C430 | ESBL | S | 1 | S | ≦0.5 | S | None | ColRNAI_1,Col(BS512)_1,Col156_1,IncFII(pRSB107)_1_pRSB107,IncFIB(AP001918)_1,IncFIA_1,IncFII_1,ColRNAI_1 |
| C432 | Non ESBL | R | 32 | R | >32 | S | None | IncFIB(AP001918)_1,Col440I_1,Col(BS512)_1,IncFII(pRSB107)_1_pRSB107,IncA/C2_1,IncFII_1,IncI_Gamma_1 |
| C439 | ESBL | R | 32 | R | >32 | S | Strong | Col(BS512),IncFIA,IncFIB(AP001918),IncFII(pAMA1167-NDM-5),IncQ1 |
| C465 | ESBL | R | 32 | R | >32 | I | None | IncFIA_1,IncFIB(AP001918)_1,IncFII(pAMA1167-NDM-5)_1_pAMA1167-NDM-5,Col(BS512)_1 |
| C470 | ESBL | R | 32 | R | >32 | S | Weak | IncFIB(AP001918)_1, IncFIA_1, IncFII(pRSB107)_1_pRSB107, Col(BS512)_1, Col440I_1 |
| C476 | ESBL | R | 32 | R | >32 | R | None | Col(BS512)_1, IncFIA_1, IncFIB(AP001918)_1, IncFII(pAMA1167-NDM-5)_1_pAMA1167-NDM-5, |
| C478 | ESBL | R | 32 | R | >32 | S | Weak | IncFIC(FII)_1,Col(BS512)_1,IncFIA_1 |
| C495 | ESBL | R | 32 | R | >32 | R | None | IncY_1,Col440I_1,Col440II_1,Col(BS512)_1,IncFIB(AP001918)_1,IncFIA_1,IncFII(pAMA1167-NDM-5)_1_pAMA1167-NDM-5 |
| C545 | ESBL | R | 32 | R | >32 | S | Weak | Col440I_1,Col(MG828)_1,IncFIA_1,IncFII(pRSB107)_1_pRSB107,Col(BS512)_1,IncFIB(AP001918)_1 |
| C547 | ESBL | R | 32 | R | >32 | S | Weak | Col440I_1,Col(BS512)_1,IncFIB(AP001918)_1,IncFII(pRSB107)_1_pRSB107,IncFIA_1,Col(MG828)_1 |
| C555 | Non ESBL | S | 0.25 | S | ≦0.5 | S | None | IncFIB(AP001918)_1,IncFIC(FII)_1,IncN2_1,ColRNAI_1 |
| C566 | ESBL | R | 32 | R | >32 | R | Strong | IncFIA_1,IncFIB(AP001918)_1,Col(BS512)_1,IncFII(pAMA1167-NDM-5)_1_pAMA1167-NDM-5 |
| C579 | ESBL | R | 32 | R | >32 | S | Strong | IncFIA_1,IncFIB(AP001918)_1,IncN2_1,IncFII(pAMA1167-NDM-5)_1_pAMA1167-NDM-5,Col(BS512)_1 |
| C584 | ESBL | R | 32 | R | >32 | R | None | IncY_1,IncFII(pAMA1167-NDM-5)_1_pAMA1167-NDM-5,IncFIB(AP001918)_1,Col(BS512)_1,IncFIA_1,IncX4_1 |
| C594 | ESBL | R | 32 | R | >32 | R | Weak | IncFIB(AP001918)_1, IncFIA_1, Col(BS512)_1, IncFII(pAMA1167-NDM-5)_1_pAMA1167-NDM-5, Col440I_1, Col440II_1 |
| C671 | ESBL | R | 32 | R | >32 | R | None | IncY_1, Col(BS512)_1, IncFIA_1, IncFII(pAMA1167-NDM-5)_1_pAMA1167-NDM-5, IncFIB(AP001918)_1, IncX4_1 |
| C685 | Non ESBL | R | 32 | R | >32 | S | None | IncFIB(AP001918)_1,IncN2_1, IncX1_1,IncFIA_1,IncFIB(pHCM2)_1_pHCM2 |
| C687 | ESBL | R | 32 | R | >32 | S | None | IncI_Gamma_1,IncFII_1,IncFII(pRSB107)_1_pRSB107,Col440I_1,IncFIB(AP001918)_1 |
| C689 | ESBL | R | 32 | R | >32 | R | Strong | IncFIB(AP001918)_1,IncFIA_1,Col(BS512)_1,IncFII(pAMA1167-NDM-5)_1_pAMA1167-NDM-5 |
| C701 | ESBL | R | 32 | R | >32 | R | Strong | IncFIB(AP001918)_1,Col(BS512)_1,IncFII(pAMA1167-NDM-5)_1_pAMA1167-NDM-5,IncFIA_1 |
| C703 | ESBL | R | 32 | R | 24 | S | None | IncY_1,Col(BS512)_1,IncFII(pAMA1167-NDM-5)_1_pAMA1167-NDM-5,IncFIA_1,Col440II_1,Col440I_1,IncFIB(AP001918)_1 |
| C705 | Non ESBL | R | 32 | R | >32 | S | Weak | IncFII_1,IncFII(pRSB107)_1_pRSB107,ColRNAI_1,IncFIA_1,IncFIB(AP001918)_1 |
| C713 | ESBL | R | 32 | R | >32 | R | Weak | IncFII(pAMA1167-NDM-5)_1_pAMA1167-NDM-5, IncFIA_1, Col(BS512)_1, IncFIB(AP001918)_1 |
| C720 | Non ESBL | R | 32 | R | >32 | S | Moderate | IncFII_1, IncA/C2_1, IncFIA_1, IncFIB(pB171)_1_pB171 |
| C725 | ESBL | R | 32 | R | >32 | R | None | Col(BS512)_1,IncFIB(AP001918)_1,IncFIA_1 |
| C727 | ESBL | R | 32 | R | >32 | S | None | Col(IRGK)_1,Col(BS512)_1,IncFIB(AP001918)_1,IncFIA_1 |
| C781 | Non ESBL | R | 32 | R | >32 | R | Strong | IncFII(pAMA1167-NDM-5)_1_pAMA1167-NDM-5,IncFIB(AP001918)_1,IncFIA_1,Col(BS512)_1 |
| C783 | ESBL | R | 32 | R | >32 | R | Strong | IncFIB(AP001918)_1,IncFIA_1,IncFII(pAMA1167-NDM-5)_1_pAMA1167-NDM-5 |
| C786 | ESBL | R | 2 | S | 0.125 | S | Weak | IncFIA_1,Col(BS512)_1,IncFIB(AP001918)_1,IncFII(pAMA1167-NDM-5)_1_pAMA1167-NDM-5 |
| C810 | ESBL | R | 32 | R | >32 | S | Weak | IncFIA_1,IncA/C2_1,IncFIB(AP001918)_1,Col(BS512)_1,IncFII(pAMA1167-NDM-5)_1_pAMA1167-NDM-5 |
| C812 | Non ESBL | R | 16 | R | >32 | R | Strong | IncFIA_1,Col(BS512)_1,IncFII(pAMA1167-NDM-5)_1_pAMA1167-NDM-5,IncFIB(AP001918)_1 |
| C827 | ESBL | R | 32 | S | 0.5 | R | Weak | IncFIB(AP001918)_1,IncFIA_1,Col(BS512)_1,IncFII(pAMA1167-NDM-5)_1_pAMA1167-NDM-5 |
| C835 | Non ESBL | R | 32 | S | 1.5 | R | Weak | IncFII(pAMA1167-NDM-5)_1_pAMA1167-NDM-5,IncFIA_1,Col(BS512)_1,IncFIB(AP001918)_1 |
| C104B | ESBL | R | 32 | R | >32 | S | None | IncFIB(AP001918)_1,IncFIA_1,IncFII(pAMA1167-NDM-5)_1_pAMA1167-NDM-5 |
| C431W | Non ESBL | R | 32 | R | >32 | S | None | Col156_1,IncI_Gamma_1,IncFIA_1,IncFIB(AP001918)_1,IncFII(pRSB107)_1_pRSB107 |
| C723B | ESBL | S | 0.125 | S | ≦0.5 | S | Moderate | IncA/C2_1,IncFIB(AP001918)_1,IncY_1,IncFII_1,IncFII_1 |
| C785B | ESBL | R | 32 | R | >32 | S | Moderate | Col(BS512)_1,Col440II_1,Col440I_1,IncFIB(AP001918)_1,IncFIA_1,IncFII(pAMA1167-NDM-5)_1_pAMA1167-NDM-5 |
| C172 | ESBL | R | 32 | R | 12 | S | Weak | IncX3_1, IncFII(pAMA1167-NDM-5)_1_pAMA1167-NDM-5, IncFIB(AP001918)_1, IncFIA_1 |
| C330 | ESBL | R | 32 | R | >32 | S | Weak | IncFIA_1,IncFIB(AP001918)_1,IncN2_1,Col156_1,Col156_1,IncFII(pRSB107)_1_pRSB107 |
| C416 | ESBL | R | 32 | R | >32 | S | None | IncY_1,IncFIA_1,IncFII(pAMA1167-NDM-5)_1_pAMA1167-NDM-5,Col(IMGS31)_1,Col(BS512)_1,IncFIB(AP001918)_1 |
| C467 | Non ESBL | R | 32 | R | >32 | S | None | IncI_Gamma_1,IncFII(pRSB107)_1_pRSB107,IncA/C2_1,Col440I_1,IncFIB(AP001918)_1 |
| C684 | ESBL | R | 32 | R | >32 | R | Weak | IncFIA_1,IncFII(pAMA1167-NDM-5)_1_pAMA1167-NDM-5,IncFIB(AP001918)_1,Col440I_1,Col440II_1,Col(BS512)_1 |
| C779 | ESBL | R | 32 | R | >32 | R | Strong | IncFIB(AP001918)_1,IncFIA_1,Col(BS512)_1,IncFII(pAMA1167-NDM-5)_1_pAMA1167-NDM-5 |
| AMR0177 | ESBL | R | 32 | R | >32 | S | None | IncFII(pAMA1167-NDM-5)_1_pAMA1167-NDM-5, Col(BS512)_1, IncFIB(AP001918)_1, IncFIA_1, IncA/C2_1 |
| AMR0262 | ESBL | R | 32 | R | >32 | I | Weak | IncFIA_1, ColRNAI_1, Col(BS512)_1, ColRNAI_1, IncFIB(AP001918)_1, IncFII(pAMA1167-NDM-5)_1_pAMA1167-NDM-5 |
| C155 | Non ESBL | R | 32 | R | 24 | R | None | IncFIA_1,IncX3_1,IncA/C2_1,IncFIB(AP001918)_1,IncFII(pAMA1167-NDM-5)_1_pAMA1167-NDM-5 |
| C440 | ESBL | R | 32 | R | 16 | S | Weak | IncFIB(AP001918)_1,IncFIA_1,IncFII(pAMA1167-NDM-5)_1_pAMA1167-NDM-5,Col(BS512)_1,Col156_1 |
| AMR0264 | ESBL | R | 32 | R | >32 | S | None | IncFIB(AP001918)_1, IncFII(pAMA1167-NDM-5)_1_pAMA1167-NDM-5, IncFIB(AP001918)_1, Col(BS512)_1,IncFIA_1,Col(BS512)_1, IncA/C2_1, ColRNAI_1 |
| C076 | Non ESBL | R | 32 | R | >32 | S | None | IncI_Gamma_1,IncFIB(AP001918)_1,Col440I_1,IncFII(pRSB107)_1_pRSB107,IncFII_1 |
| AMR0265 | Non ESBL | R | 32 | R | >32 | S | None | Col(MG828)_1,ColRNAI_1,IncFIA_1,IncI_Gamma_1,IncFIC(FII)_1 |
| AMR0279 | ESBL | R | 32 | R | >32 | S | Moderate | ColpVC_1,IncFIB(pQil)_1_pQil,IncFIA(HI1)_1_HI1,IncFIB(K)_1_Kpn3,IncFIC(FII)_1,ColRNAI_1,Col440I_1,IncX1_1,IncFII_1_pKP91,IncR_1 |
| C366 | ESBL | R | 32 | R | >32 | S | Strong | IncFIB(AP001918)_1,Col(MG828)_1,IncI2_1_Delta,IncFIA_1,Col156_1,IncFII(pRSB107)_1_pRSB107,Col8282_1 |
| C425 | ESBL | R | 32 | R | 24 | I | None | IncFIB(AP001918)_1, IncFIA_1, Col(BS512)_1, IncFII(pAMA1167-NDM-5)_1_pAMA1167-NDM-5 |
| C691 | ESBL | R | 32 | R | >32 | S | None | IncFIB(AP001918)_1,IncFII(pAMA1167-NDM-5)_1_pAMA1167-NDM-5,Col440I_1,Col440II_1,IncFIA_1,IncY_1,Col(BS512)_1 |
| C428 | ESBL | R | 32 | R | >32 | S | Weak | Col440I_1, IncFIB(AP001918)_1, IncFIA_1, IncFII(pRSB107)_1_pRSB107, Col(BS512)_1, Col(MG828)_1, ColRNAI_1 |
| C150 | ESBL | R | 32 | R | >32 | S | None | IncFII(pAMA1167-NDM-5)_1_pAMA1167-NDM-5,IncX3_1,IncFIB(AP001918)_1,IncB/O/K/Z_2,IncFIA_1,IncI1_1_Alpha |
| C299 | ESBL | R | 32 | R | >32 | S | Weak | IncFIB(AP001918)_1,IncFIA_1,IncFII(pRSB107)_1_pRSB107,Col(MG828)_1,Col440I_1,Col(BS512)_1,ColRNAI_1 |
| AMR0278 | ESBL | R | 32 | R | >32 | S | None | ColKP3,IncC,IncFIA,IncFIB(AP001918),IncFII(pAMA1167-NDM-5),IncL,IncX3 |

Abbreviation. ESBL, extended-spectrum-lactamases; CPFX, ciprofloxacin; LVFX, levofloxacin; NFT, nitrofurantoin; R, resistance; I, intermediate; S, sensitivity

**Table S6.** Relationship between biofilm formation ability and carriage of any adhesin genes.

| Virulence factors | Biofilm-forming^a^  (n = 29) | Non-biofilm-forming^b^  (n = 74) | *p-value* |
| --- | --- | --- | --- |
| *fim* | 29 | 74 | -^c^ |
| *ecp* | 28 | 73 | 1 |
| *csg* | 29 | 74 | -^c^ |
| *eae* | 24 | 57 | 0.075 |
| *pap* | 8 | 14 | 0.059 |
| *sfa* | 0 | 0 | -^c^ |
| *afa,draP* | 1 | 3 | 0.572 |
| *aap* | 1 | 0 | -^c^ |
| *cfa* | 2 | 5 | 0.691 |
| *aaf* | 0 | 3 | 0.557 |
| *fae* | 1 | 1 | 1 |
|  |  |  |  |

^a^Biofilm-forming was characterized based on medium to strong biofilm formation.

^b^Non-biofilm forming was characterized based on non to weak biofilm formation.

^c^Symbol; -, No statistics are computed because this gene are constants.

**Table S7.** Antimicrobial resistance determinants of FQ-CREc urine isolates in Thailand.

| **MLST** | **Clermont phylogroup** | ***bla*_NDM-type_** | ***bla*_OXA-type_** | ***bla*_CTX-M-type_** | ***bla*_TEM-type_** | ***bla*_CMY-type_** | ***bla*_VEB-type_** | ***qnr*** | ***aac(6)-Ib-cr*** | ***gyrA*** | ***gyrB*** | ***parC*** | ***parE*** | **ESBL** | **CPFX** | **LVFX** | **NFT** | **Biofilm** | **Profiles** | **N** |
| --- | --- | --- | --- | --- | --- | --- | --- | --- | --- | --- | --- | --- | --- | --- | --- | --- | --- | --- | --- | --- |
| 410 | C | NDM-5 | - | CTX-M-15 | TEM-1B | CMY-2 | - | - | - | + | - | + | + | + | R | R | R | Strong biofilm | 1 | 9 |
| 410 | C | NDM-5 | OXA-1 | CTX-M-15 | TEM-1B | CMY-2 | - | - | + | + | - | + | + | + | R | R | R | non biofilm producers | 2 | 7 |
| 410 | C | NDM-5 | OXA-1 | CTX-M-15 | TEM-1B | CMY-2 | - | - | + | + | - | + | + | + | R | R | S | non biofilm producers | 3 | 6 |
| 410 | C | NDM-5 | OXA-1 | CTX-M-15 | TEM-1B | CMY-2 | - | - | + | + | - | + | + | + | R | R | R | weak biofilm | 4 | 3 |
| 410 | C | NDM-5 | OXA-1 | CTX-M-15 | TEM-1B | CMY-2 | - | - | + | + | - | - | + | + | R | R | R | non biofilm producers | 5 | 2 |
| 410 | C | NDM-5 | OXA-1 | CTX-M-15 | TEM-1B | CMY-2 | - | - | + | + | - | - | + | + | R | R | I | non biofilm producers | 6 | 2 |
| 410 | C | NDM-5 | OXA-1 | CTX-M-15 | TEM-1B | CMY-2 | - | - | + | + | - | + | - | + | R | R | R | non biofilm producers | 7 | 1 |
| 410 | C | NDM-5 | OXA-1 | CTX-M-15 | TEM-1B | CMY-2 | - | *qnrS*1 | + | + | - | + | + | + | R | R | S | non biofilm producers | 8 | 1 |
| 410 | C | NDM-5 | OXA-1 | CTX-M-15 | TEM-1B | CMY-2 | - | - | + | + | - | + | + | + | R | R | R | Strong biofilm | 9 | 1 |
| 410 | C | NDM-5 | OXA-1 | CTX-M-15 | TEM-1B | CMY-2 | - | - | - | + | - | + | + | + | R | R | I | Strong biofilm | 10 | 1 |
| 410 | C | NDM-5 | OXA-1 | CTX-M-15 | TEM-1B | CMY-2 | - | - | - | + | - | + | + | + | R | R | R | non biofilm producers | 11 | 1 |
| 410 | C | NDM-5 | - | CTX-M-15 | TEM-1B | CMY-2 | - | *-* | - | + | - | + | + | + | R | R | S | weak biofilm | 12 | 1 |
| 410 | C | NDM-5 | OXA-1 | CTX-M-15 | TEM-1B | CMY-2 | - | - | + | + | - | + | + | + | R | R | S | weak biofilm | 13 | 1 |
| 410 | C | NDM-5 | OXA-1 | CTX-M-15 | TEM-1B | CMY-2 | - | *-* | + | + | - | + | + | - | R | R | R | Strong biofilm | 14 | 1 |
| 410 | C | NDM-5 | - | CTX-M-15 | TEM-1B | CMY-2 | - | - | - | + | - | + | + | + | R | R | R | weak biofilm | 15 | 1 |
| 410 | C | NDM-5 | OXA-1 | CTX-M-15 | TEM-1B | CMY-2 | - | - | + | + | - | + | + | - | R | R | R | weak biofilm | 16 | 1 |
| 410 | C | NDM-5 | OXA-1 | CTX-M-15 | TEM-1B | CMY-2 | - | - | + | + | - | + | + | + | R | R | S | moderate biofilm | 17 | 1 |
| 410 | C | NDM-5 | OXA-1 | CTX-M-15 | TEM-1B | CMY-2 | - | - | + | + | - | + | + | + | R | R | I | weak biofilm | 18 | 1 |
| 410 | C | NDM-5 | OXA-1, OXA-10 | CTX-M-15 | TEM-1B | CMY-2 | - | - | + | + | - | + | + | + | R | R | S | non biofilm producers | 19 | 1 |
| 410 | C | NDM-5 | OXA-1, OXA-181 | CTX-M-15 | TEM-1B | CMY-2 | - | *qnrS*1 | - | + | - | + | + | + | R | R | S | non biofilm producers | 20 | 1 |
| 410 | C | NDM-1 | OXA-1 | CTX-M-15 | TEM-1B | CMY-2 | - | - | + | + | - | + | - | + | R | R | S | non biofilm producers | 21 | 1 |
| 410 | C | NDM-1 | OXA-1 | CTX-M-15 | TEM-1B | CMY-2 | - | - | + | + | - | + | + | + | R | R | I | Strong biofilm | 22 | 1 |
| 410 | C | NDM-1 | OXA-1, OXA-10 | CTX-M-15 | - | - | VEB-1 | *-* | + | + | - | + | + | + | R | R | R | non biofilm producers | 23 | 1 |
| 410 | C | NDM-1 | OXA-1 | CTX-M-15 | TEM-1B | CMY-2 | - | - | + | + | - | + | + | + | R | R | S | Strong biofilm | 24 | 1 |
| 410 | C | NDM-1 | OXA-1 | - | TEM-1B | CMY-2 | - | *-* | + | + | - | + | + | + | R | R | S | Strong biofilm | 25 | 1 |
| 410 | C | NDM-1 | OXA-1 | - | TEM-1B | - | - | *-* | + | + | - | + | + | - | R | R | S | non biofilm producers | 26 | 1 |
| 410 | C | NDM-1 | OXA-1, OXA-10 | CTX-M-15 | TEM-1B | CMY-2 | VEB-1 | *qnrA1* | + | + | - | + | + | + | R | R | S | non biofilm producers | 27 | 1 |
| 410 | C | NDM-1 | - | CTX-M-15 | - | - | - | *qnrB17,qnrS1* | + | + | - | + | + | + | R | R | S | moderate biofilm | 28 | 1 |
| 410 | C | - | OXA-1, OXA-181 | CTX-M-15 | TEM-1B | CMY-2 | - | *qnrS1* | + | + | - | + | + | + | R | R | S | non biofilm producers | 29 | 1 |
| 405 | D | NDM-5 | OXA-1 | CTX-M-15 | TEM-1B | - | - | - | + | + | + | + | + | + | R | R | S | weak biofilm | 1 | 2 |
| 405 | D | NDM-5 | OXA-1 | CTX-M-15 | TEM-1B | - | - | - | + | + | + | + | + | + | R | R | S | non biofilm producers | 2 | 2 |
| 405 | D | NDM-5 | - | CTX-M-15 | TEM-1B | - | - | - | - | + | + | + | + | + | R | R | S | weak biofilm | 3 | 2 |
| 405 | D | NDM-5 | - | CTX-M-15 | TEM-1B | - | - | - | - | + | + | + | + | + | R | R | S | non biofilm producers | 3 | 1 |
| 405 | D | NDM-3 | OXA-1 | CTX-M-15 | TEM-1B | - | - | - | + | + | + | + | + | + | R | R | S | Strong biofilm | 4 | 1 |
| 405 | D | NDM-5 | OXA-1 | CTX-M-15 | TEM-1B | - | - | - | + | + | + | - | + | + | R | R | S | weak biofilm | 5 | 1 |
| 405 | D | NDM-5 | OXA-1 | CTX-M-15 | - | - | - | - | + | + | + | + | + | + | R | R | S | weak biofilm | 6 | 1 |
| 405 | D | NDM-5 | - | - | TEM-1B | CMY-42 | - | - | - | + | + | - | + | - | R | R | S | non biofilm producers | 7 | 1 |
| 405 | D | NDM-3 | OXA-1 | CTX-M-15 | TEM-1B | - | - | - | + | + | + | + | + | + | R | R | S | weak biofilm | 8 | 1 |
| 405 | D | NDM-5 | - | CTX-M-15 | - | - | - | - | - | + | + | - | + | + | R | R | S | weak biofilm | 9 | 1 |
| 361 | A | NDM-5 | - | - | TEM-1B | CMY-42 | - | - | - | + | - | + | - | - | R | R | S | non biofilm producers | 1 | 5 |
| 361 | A | NDM-5 | - | - | - | CMY-42 | - | - | - | + | - | + | - | - | R | R | S | non biofilm producers | 2 | 3 |
| 361 | A | NDM-5 | OXA-1 | - | TEM-1B | CMY-42 | - | - | + | + | - | + | - | - | R | R | S | non biofilm producers | 3 | 1 |
| 361 | A | NDM-5 | - | - | TEM-1B | CMY-42 | - | - | - | + | + | + | - | + | R | R | S | non biofilm producers | 4 | 1 |
| 361 | A | NDM-1 | OXA-1 | - | TEM-1B | CMY-42 | - | - | + | + | - | + | - | - | R | R | S | non biofilm producers | 5 | 1 |
| 361 | A | NDM-5 | - | - | - | CMY-42 | - | - | - | + | - | - | - | - | R | R | S | non biofilm producers | 6 | 1 |
| 167 | A | NDM-5 | OXA-1 | CTX-M-15 | TEM-1B | - | - | - | - | + | - | + | + | + | R | R | S | weak biofilm | 1 | 1 |
| 167 | A | NDM-5 | - | CTX-M-55 | TEM-1B | - | - | *qnrS1* | - | + | - | + | + | + | R | R | S | Strong biofilm | 2 | 1 |
| 167 | A | NDM-5 | OXA-140 | CTX-M-15 | TEM-1B | - | - | - | - | + | - | + | + | + | R | R | S | weak biofilm | 3 | 1 |
| 167 | A | NDM-5 | OXA-1 | CTX-M-55 | TEM-1B | - | - | *-* | + | + | - | + | + | + | R | R | S | weak biofilm | 4 | 1 |
| 448 | B1 | NDM-4 | OXA-1 | - | TEM-1B | CMY-2 | - | - | + | + | - | + | + | + | R | R | S | weak biofilm | 1 | 1 |
| 448 | B1 | NDM-4 | OXA-1 | - | TEM-1B | CMY-2 | - | - | + | + | - | + | + | - | R | R | R | non biofilm producers | 2 | 1 |
| 448 | B1 | NDM-7 | - | CTX-M-15 | TEM-1B | - | - | - | - | + | - | + | + | + | R | R | S | weak biofilm | 3 | 1 |
| 448 | B1 | NDM-7 | - | CTX-M-15 | - | - | - | - | - | + | - | + | + | + | R | R | S | non biofilm producers | 4 | 1 |
| 354 | F | NDM-1 | - | CTX-M-24, CTX-M-55 | - | - | - | - | - | + | + | + | + | + | R | R | S | Strong biofilm | 1 | 1 |
| 354 | F | NDM-1 | - | CTX-M-24, CTX-M-55 | - | - | - | - | - | + | + | + | + | + | R | R | S | moderate biofilm | 2 | 1 |
| 354 | F | NDM-1 | OXA-1 | CTX-M-55, CTX-M-24 | TEM-150 | CMY-4 | - | *qnrB6* | - | + | + | + | + | - | R | R | S | moderate biofilm | 3 | 1 |
| 10210 | C | NDM-5 | OXA-1 | CTX-M-15 | TEM-1B | CMY-2 | - | - | + | + | - | + | + | + | R | R | S | non biofilm producers | 1 | 1 |
| 10210 | C | NDM-5 | OXA-1 | CTX-M-15 | TEM-1B | CMY-2 | - | - | + | + | - | + | + | - | R | R | R | Strong biofilm | 2 | 1 |
| 10210 | C | NDM-5 | OXA-1 | CTX-M-15 | TEM-1B | CMY-2 | - | - | + | + | - | - | - | + | R | R | S | non biofilm producers | 3 | 1 |
| 46 | A | NDM-5 | OXA-1 | CTX-M-15 | TEM-1C | - | - | - | + | + | - | + | + | + | R | R | S | non biofilm producers | 1 | 1 |
| 46 | A | NDM-5 | OXA-1 | CTX-M-15 | TEM-1B | - | - | - | + | + | - | + | + | - | R | R | S | weak biofilm | 2 | 1 |
| 38 | D | NDM-1 | OXA-1 | CTX-M-27 | TEM-1B | CMY-4 | - | - | + | + | - | + | - | + | S | S | S | moderate biofilm | 1 | 1 |
| 38 | D | NDM-1 | - | CTX-M-27 | - | - | - | - | - | + | - | + | + | + | R | R | S | weak biofilm | 2 | 1 |
| 131 | B2 | NDM-5 | OXA-1 | CTX-M-15 | TEM-1B | - | - | - | + | + | - | + | + | + | R | R | S | moderate biofilm | 1 | 1 |
| 131 | B2 | - | - | CTX-M-27 | - | - | - | - | - | + | - | + | + | + | R | R | S | Strong biofilm | 2 | 1 |
| 2011 | F | NDM-1 | OXA-1 | CTX-M-15 | TEM-1B | CMY-4 | - | - | + | + | + | + | + | - | R | R | S | moderate biofilm | 1 | 1 |
| 457 | F | NDM-1 | - | - | TEM-1B | - | - | - | - | + | + | + | + | + | R | R | S | moderate biofilm | 1 | 1 |
| 1193 | B2 | NDM-1 | OXA-10 | - | - | - | - | - | - | + | - | + | + | - | R | R | S | moderate biofilm | 1 | 1 |
| 1702 | A | NDM-5 | - | - | - | CMY-42 | - | - | - | + | - | + | + | - | R | R | S | non biofilm producers | 1 | 1 |
| 34 | A | NDM-5 | OXA-1 | CTX-M-15 | - | - | - | - | + | + | - | - | - | + | S | S | S | non biofilm producers | 1 | 1 |
| 88 | C | NDM-1 | - | - | TEM-1B | - | - | - | - | + | + |  | + | - | S | S | S | non biofilm producers | 1 | 1 |

Abbreviation. MLST, multilocus sequence typing; extended-spectrum-lactamases; CPFX, ciprofloxacin; LVFX, levofloxacin; NFT, nitrofurantoin; +, positive; -, negative; R, resistance; S, sensitivity
